# Co-occurrence of multiple pathologies in a case of frontotemporal dementia with TBK1 mutation: first in vivo detection of alpha-synuclein and tau co-pathology

**DOI:** 10.1186/s40478-025-02081-1

**Published:** 2025-07-25

**Authors:** Alexander M. Bernhardt, Sigrun Roeber, Viktoria Ruf, Elisabeth Wlasich, Endy Weidinger, Sebastian Longen, Svenja V. Trossbach, Johannes Gnörich, Matthias Brendel, Jochen Herms, Armin Giese, Günter U. Höglinger, Johannes Levin

**Affiliations:** 1 Department of Neurology, Ludwig-Maximilians-Universität München, München, Germany; 2 German Center for Neurodegenerative Diseases (DZNE), site Munich, Germany; 3 Center for Neuropathology and Prion Research, Faculty of Medicine, Ludwig-Maximilians- University Munich, Munich, Germany; 4 Munich Cluster for Systems Neurology (SyNergy), Munich, Germany; 5 Aesku.Diagnostics GmbH, Wendelsheim, Germany; 6 MODAG GmbH, Wendelsheim, Germany; 7 Department of Nuclear Medicine, LMU University Hospital, LMU Munich, Munich, Germany

## Abstract

We present the case of a 74-year-old woman with behavioral variant frontotemporal dementia (bvFTD) linked to a pathogenic TANK-binding kinase 1 (TBK1) mutation (c.1349_1352del; p.Ile450Lysfs*15). During clinical workup, the patient underwent comprehensive biomarker analysis, including tau positron emission tomography (PET) and cerebrospinal fluid (CSF) seed amplification assay (SAA) for α-synuclein (αSyn). While CSF biomarkers for Alzheimer’s disease were normal, the αSyn SAA was clearly positive, indicating misfolded αSyn aggregates. Tau PET revealed increased [^18^F]PI-2620 uptake in the basal ganglia. Genetic testing confirmed autosomal dominant TBK1-associated FTD. The patient’s condition deteriorated over the following year, with rapid cognitive decline and the emergence of cortical signs. Post-mortem neuropathological analysis confirmed multiple proteinopathies: FTLD-TDP43 (subtype A), Lewy body disease (limbic type, Braak stage 5), argyrophilic grain disease (AGD), aging-related tau astrogliopathy (ARTAG), and primary age-related tauopathy (PART). This is the first reported TBK1-FTD case with in vivo detection of αSyn pathology via SAA and in vivo monitoring of tau pathology. The case expands the clinical and neuropathological spectrum of TBK1-associated FTD. Our findings support a broader interpretation of TBK1-associated neurodegeneration and highlight the importance of multimodal diagnostic approaches that integrate molecular, genetic, imaging, and neuropathological tools. This case also underscores the utility of αSyn SAA and tau PET in detecting co-pathologies that may otherwise remain clinically silent and illustrates the need for further studies exploring the molecular cross-talk between TBK1, tau, and αSyn pathologies.

## Introduction

We report a 74-year-old female with frontotemporal dementia (FTD) linked to a pathogenic TANK-binding kinase 1 (TBK1) mutation (c.1349_1352del (p.Ile450Lysfs*15)). At autopsy, this case featured multiple neurodegenerative pathologies: frontotemporal lobar degeneration with TDP-43 proteinopathy (FTLD-TDP-43), Lewy-body disease (LBD), argyrophilic grain disease (AGD) and Primary Age-Related Tauopathy (PART). This is the first documented FTD case due to a TBK-1 mutation with in vivo detection of Lewy-body co-pathology using an α-synuclein (αSyn) seed amplification assay (SAA), alongside in vivo longitudinal tau pathology monitoring using tau PET.

## Case presentation

At diagnosis, the patient, a right-handed 74-year-old woman, was assessed following a two-year history of behavioral changes including increased apathy, neglect of self-care, and substance use, consistent with behavioral variant FTD [1]. Motor function was intact with no signs of upper motor neuron disease or extrapyramidal/parkinsonian features (no rigidity, tremor, or bradykinesia).

Neuropsychological testing including the Consortium to Establish a Registry for Alzheimer’s Disease (CERAD) battery showed normal processing speed and attention, slight backward digit span impairment, and significant deficits in verbal learning, though recognition remained intact. Executive functions were mildly to moderately impaired, especially in inhibition and flexibility, with increased impulsivity. Language and visuo-constructive skills were within normal limits (see Table 1 for full scores).

**Table 1:** Neuropsychological test results at baseline and one-year follow-up. Summary of raw scores and corresponding Z-scores (or percentile ranks) for key cognitive domains, demonstrating progression of memory, executive, language, and visuospatial impairments over one year. N/A indicates that the test was not administered at follow-up because the patient was too cognitively impaired to complete it. Word list recognition Z-score at follow‐up could not be calculated due to false positives. Abbreviations: WMS-R = Wechsler Memory Scale-Revised

Test	Baseline	One-Year Follow-Up
Mini-Mental State Examination (MMSE)	25/30	24/30
Word List Learning	13 (3/5/5) (Z-score=-2.64)	7 (1/3/3) (Z-score=-4.49)
Word List Recall	5 (Z-score=-1.21)	1/10 (Z-score=-3.18)
Word List Savings	100% (Z-score = 0.93)	34% (Z-score=-2.71)
Word List Intrusions	1 (Z-score=-0.82)	7 (Z-score=-2.61)
Word List Recognition	10 correct, 0 false positives (Z-score = 0.68)	7/10 correct, 2 false positives
Discriminability	100%	75% (Z-score=-3.00)
Constructional Praxis	11 (Z-score=-1.35)	10 (Z-score=-0.36)
Constructional Praxis Recall	8 (Z-score=-0.13)	3 (Z-score=-2.03)
Constructional Praxis Savings	73% (Z-score=-0.43)	30% (Z-score=-1.97)
Semantic Fluency (Animals)	17 (Z-score=-0.59)	11 (Z-score=-1.82)
Phonemic Fluency (S-words)	8 (Z-score=-0.29)	6 (Z-score=-1.35)
Naming (Boston Naming Test − 15 items)	14/15 (Z-score = 0.13)	12/15 (Z-score=-1.24)
Trail-Making Test A	43 s (Z-score = 0.31)	61 s (Z-score=-0.76)
Trail-Making Test B	98 s (Z-score = 0.56)	N/A
Trail-Making Test B/A	2.3 (Z-score=-0.31)	N/A
Memory: Digit Span (WMS-R) - forward	5 (Percentile Rank = 15)	N/A
Memory: Digit Span (WMS-R) - backward	3 (Percentile Rank = 5)	N/A
Executive Function: Color-Word Interference Test (Stroop Test) - processing speed (III)	31 s (Percentile Rank = 27–35)	N/A
Color-Word Interference Test (Stroop Test) - interference condition (III)	76 s (Percentile Rank = 9–13)	N/A
Color-Word Interference Test (Stroop Test) - interference control (III-II)	46 s (Percentile Rank = 9–13)	N/A

**Fig. 1:**
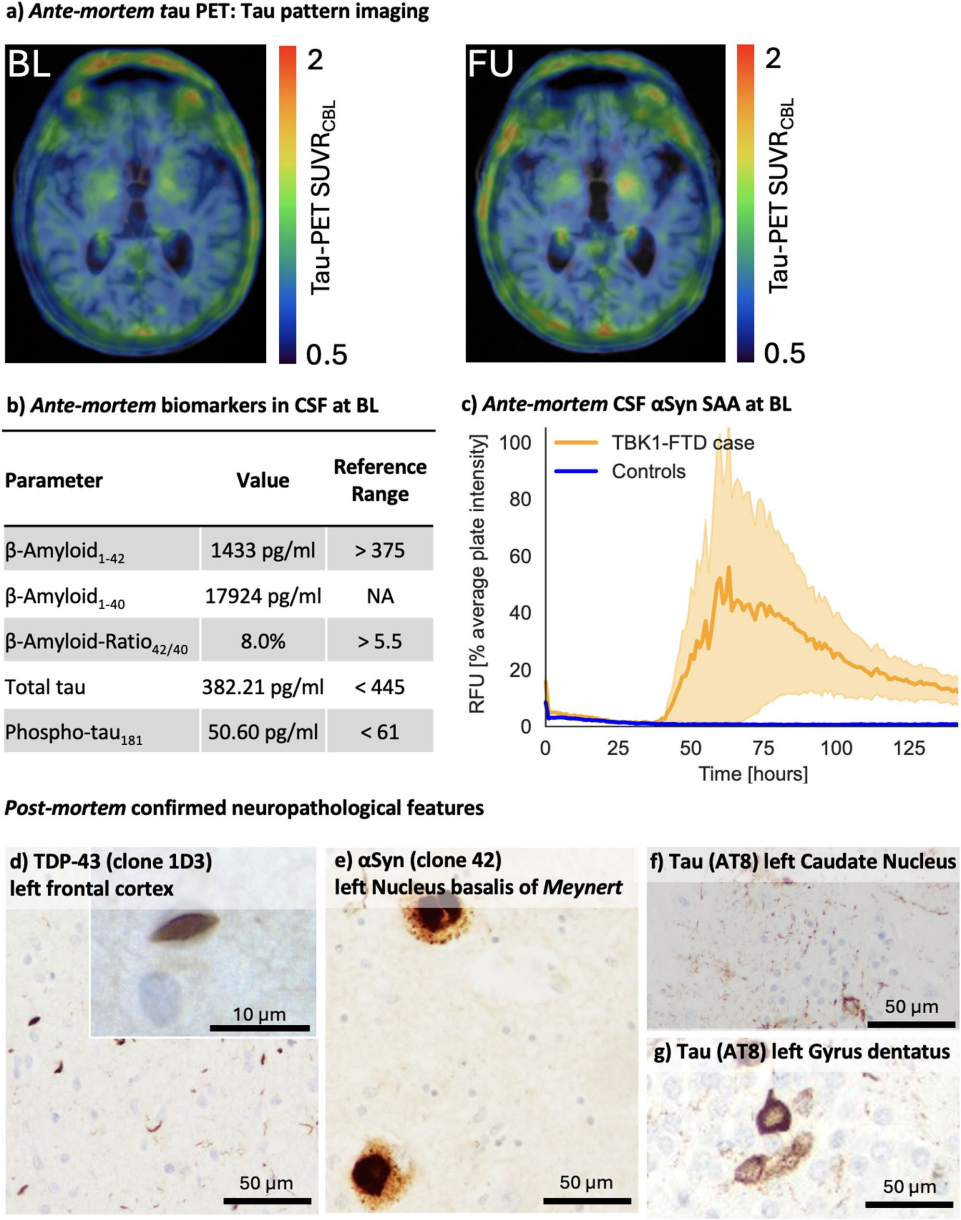
*Ante-mortem* detection of *post-mortem* confirmed pathologies as well as immunohistochemistry of presented case. **(a)** Ante-mortem tau PET at baseline (BL) and one year later at follow-up (FU): tau pattern imaging with standard uptake value ratios (SUVR). **(b)** *Ante-mortem* biomarkers in cerebrospinal fluid (CSF) at diagnosis indicating the absence of Alzheimer pathology **(c)** *Ante-mortem* CSF αSyn Seed Amplification Assay (SAA) at BL indicating the presence of Lewy-body (LB) pathology in the TBK1-FTD case (yellow curve) versus absence of signal in controls without clinical and biomarker evidence of neurodegenerative disease (blue curve, *n* = 20). Relative fluorescence unit (RFU) values are normalized to the maximum intensity of fluorescence of the respective experimental plate. Each curve represents the average of the fours replicates ± standard error of the mean. **(d)** TDP-43 pathology (clone 1D3) of the left frontal cortex **(e)** Moderate number of Lewy bodies and Lewy neurites (clone 42) in the left Nucleus basalis of *Meynert* **(f)** AT8 positive, mainly glial tau aggregates of the left Caudate Nucleus **(g)** AT8 positive, neuronal tau aggregates in the left Gyrus dentatus consistent with argyrophilic grain disease (AGD) pathology

Cerebrospinal fluid (CSF) analysis was performed using the INNOTEST^®^ ELISA platform (Fujirebio Europe N.V., Belgium) out of clinical routine CSF examination, revealing normal β-amyloid, phosphorylated tau_181_, and total tau levels. A positive αSyn SAA indicated the presence of misfolded aSyn aggregates suggestive of Lewy-body pathology. Using our previously validated αSyn SAA [2], CSF from 20 age- and sex-matched individuals without any clinical or biomarker evidence of neurodegenerative disease showed no seeding activity. Other CSF parameters were normal, with no evidence of infection or inflammation. The initial tau PET scan at diagnosis revealed increased [^18^F]PI-2620 tracer uptake in the basal ganglia, particularly on the left side with a quantitative standard uptake value ratio SUVR of 1.28, while cortical areas did not exhibit significant [^18^F]PI-2620 tracer uptake (Fig. 1a). Genetic analysis used a targeted dementia/FTD-ALS gene panel (APOE, APP, CHMP2B, CSF1R, DNAJC5, DNMT1, EPM2A, GRN, ITM2B, MAPT, NHLRC1, NOTCH3, OPTN, PRNP, PSEN1, PSEN2, SQSTM1, TARDBP, TBK1, TREM2, TUBA4A, TYROBP, UBQLN2, VCP), plus C9orf72 repeat expansion and APOE4 genotyping. This identified a pathogenic TBK1 mutation (c.1349_1352del (p.Ile450Lysfs*15)), consistent with autosomal dominant inheritance and resulting in TBK1 haploinsufficiency [3] - and revealed no other pathogenic or likely pathogenic variants. The patient’s three-year-younger sister was also diagnosed with a behavioral variant FTD carrying the same pathogenic TBK1 mutation, while no other family members had a history of neurodegenerative diseases.

One year later, the patient exhibited marked cognitive decline, dysphagia, and significant weight loss (BMI 17.0). Neurological assessment showed brisk symmetric reflexes and ideomotor apraxia, indicative of cortical dysfunction. No parkinsonian features (rigidity, tremor, bradykinesia) emerged. At one-year follow‐up, neuropsychological reassessment documented decline across all cognitive domains. Verbal memory was severely reduced: learning and delayed recall scores were markedly lower, and recognition accuracy decreased substantially. Both semantic and phonemic fluencies were significantly lower. Executive tasks requiring cognitive flexibility, inhibition, and working memory exhibited pronounced slowing, increased errors, and failure to complete certain measures due to cognitive fatigue. Visuo‐constructive copying remained largely intact, but delayed recall of visuospatial designs was significantly impaired. These findings indicate progressive, multifocal cognitive deterioration with relative preservation of basic visuoconstruction (see Table 1). A follow-up tau PET at the one-year mark showed a slight increase in basal ganglia [^18^F]PI-2620 uptake to an SUVR of 1.32, with cortical tracer binding remaining within normal limits (Fig. 1b).

*Post-mortem* examination (three years after first symptom onset) included semiautomated immunohistochemistry on a Ventana BenchMark XT platform (Ventana/Roche, Basel, Switzerland) using primary antibodies at the following dilutions: α-synuclein (mouse monoclonal clone 42, Abcam, Cambridge, UK; 1:2,000), TDP-43 (rat monoclonal clone 1D3, in-house; 1:50), phospho-tau (AT8; mouse monoclonal pSer202/pThr205, Invitrogen/Thermo Fisher, Carlsbad, CA, USA; 1:200), RD3 (3R phospho-tau, mouse monoclonal clone 8E6/C11, Millipore; 1:800) and RD4 (four-repeat (4R) phospho-tau, mouse monoclonal clone 1E1/A6, Millipore; 1:4,000). The examination revealed:

- FTLD-TDP43 (Subtype A): Widespread TDP-43 protein aggregates, including neuronal cytoplasmic inclusions and short dystrophic neurites, primarily in the frontal and temporal cortices, with involvement of subcortical regions such as the caudate nucleus, putamen, and nucleus accumbens, ventromedial thalamus, amygdala, entorhinal cortex.
- LBD (McKeith: limbic-stage, Braak stage 5): mild αSyn pathology according to Braak-stage 5.
- Tau-pathology: neuronal and glial pathology regarding shape and distribution pattern characteristic of AGD (Saito 4), ARTAG, PART (Braak&Braak II of VI).
- No detectable β-amyloid plaques (Thal-Phase 0).

Secondary findings included mild atherosclerosis and scattered microinfarcts. A summary of the ante-mortem biomarker and imaging results alongside representative post-mortem immunohistochemical findings is shown in Fig. 1.

## Discussion and conclusions

The exploration of αSyn co-pathology in FTD is minimal. In one study [4], all FTLD-TDP-43 cases with concurrent LBD pathology had genetic mutations– specifically, two with progranulin mutations and one with a C9orf72 repeat expansion. The clinical phenotype of TBK1-associated FTD is still being elucidated, where some patients show significant early memory impairment, while others may present with extrapyramidal features or cerebellar signs– ranging from PSP-like rigidity and supranuclear gaze palsy to progressive cerebellar ataxia and imaging evidence of mesencephalic or cerebellar atrophy [5, 6]. However, the absence of motor neuron disease (MND) in the current case is noteworthy, given that TBK1 mutations are often associated with FTD-MND.

While TBK1-FTD is primarily associated with TDP-43 (FTLD (subtype B) or MND) pathologies [7], the presence of both AGD, ARTAG and PART in this case adds complexity to the clinical picture. The interpretation of increased tracer uptake, particularly in non-Alzheimer tauopathies such as AGD, ARTAG and PART, requires further investigation, as the exact clinical relevance of these pathologies is not yet fully understood. In our TBK1-FTD case, basal ganglia SUVRs of 1.28 at baseline and 1.32 at follow-up indicate a mild but measurable tau accumulation, intermediate between cognitively healthy controls and classical PSP-RS, demonstrating that [^18^F]PI-2620 can also detect subtle non-cortical tau pathologies [8]. Post-mortem AT8 immunohistochemistry confirmed tau pathology in the globus pallidus of the TBK1 carrier, but quantitative AT8 occupancy (%N/Area) was only 0.57, markedly lower than the 4.06 ± 2.45 observed in a reference cohort of seven PSP patients, consistent with a copathological rather than a primary 4R tauopathy [9]. Previous work indicates that neuronal and oligodendroglial tau deposits drive the [¹⁸F]PI-2620 PET signal more strongly than astroglial inclusions; a relatively higher proportion of astroglial tau in our case may thus underlie the lower AT8 burden despite positive PET findings. Importantly, [¹⁸F]PI-2620 exhibits high affinity for both 3/4R and 4R tau isoforms, with particular selectivity for aggregated 4R species– making it well suited to capture mixed or non-Alzheimer tau pathologies as seen here [10].

Recent studies suggest that TBK1 may influence tau phosphorylation and aggregation, in addition to its role in TDP-43 proteinopathy [11]. The co-occurrence of AGD, ARTAG and PART in this case– with the first described already by Koriath et al., 2017 - raises the question of whether TBK1 mutations could predispose to or exacerbate tau-driven neurodegeneration. Further research is needed to clarify these molecular links and their clinical implications.

This case underscores the critical need for comprehensive diagnostic approaches that integrate molecular, imaging, and neuropathological assessments. The findings suggest that TBK1 mutations may predispose patients to a multifaceted neurodegenerative process, highlighting the potential and need for personalized targeted therapeutic strategies.

## Data Availability

Due to the nature of this case report and the inclusion of detailed clinical, genetic, and imaging data, the dataset is not publicly available to protect patient confidentiality. Anonymized data may be shared by the corresponding author upon reasonable request, provided appropriate institutional and ethical approvals are in place.

## Abbreviations

4R: four–repeat
AGD: Argyrophilic grain disease
ARTAG: Aging–related tau astrogliopathy
αSyn: Alpha–synuclein
BMI: Body mass index
bvFTD: Behavioral variant frontotemporal dementia
CSF: Cerebrospinal fluid
FTD: Frontotemporal dementia
FTLD: Frontotemporal lobar degeneration
LBD: Lewy body disease
PART: Primary age–related tauopathy
PET: Positron emission tomography
SAA: Seed amplification assay
SUVR: standard uptake value ratio
TDP: 43–TAR DNA–binding protein 43
TBK1: TANK–binding kinase 1
